# Impact of Endoplasmic Reticulum Stress in Otorhinolaryngologic Diseases

**DOI:** 10.3390/ijms21114121

**Published:** 2020-06-09

**Authors:** Su Young Jung, Sung Su Kim, Seung Geun Yeo

**Affiliations:** 1 Department of Otorhinolaryngology-Head and Neck Surgery, Myongji Hospital, Hanyang University College of Medicine, Goyang 10475, Korea; monkiwh35@hanmail.net; 2 Medical Research Center for Bioreaction to Reactive Oxygen Species and Biomedical Science Institute, School of Medicine, Graduate School, Kyung Hee University, Seoul 02447, Korea; sgskim@khu.ac.kr; 3 Department of Otorhinolaryngology—Head & Neck Surgery, School of Medicine, Kyung Hee University, Seoul 02447, Korea

**Keywords:** endoplasmic reticulum stress, otorhinolaryngologic disease, otitis media, hearing loss, chronic rhinosinusitis, allergic rhinitis, obstructive sleep apnea, intermittent hypoxia

## Abstract

The endoplasmic reticulum (ER) is an important organelle for normal cellular function and homeostasis in most living things. ER stress, which impairs ER function, occurs when the ER is overwhelmed by newly introduced immature proteins or when calcium in the ER is depleted. A number of diseases are associated with ER stress, including otorhinolaryngological diseases. The relationship between ER stress and otorhinolaryngologic conditions has been the subject of investigation over the last decade. Among otologic diseases associated with ER stress are otitis media and hearing loss. In rhinologic diseases, chronic rhinosinusitis, allergic rhinitis, and obstructive sleep apnea are also significantly associated with ER stress. In this review, we provide a comprehensive overview of the relationship between ER stress and otorhinolaryngological diseases, focusing on the current state of knowledge and mechanisms that link ER stress and otorhinolaryngologic diseases.

## 1. Introduction

The endoplasmic reticulum (ER), a vesicle-like structure that branches out from the nuclear membrane, is categorized into two types: rough ER, containing ribosomes on its membrane surface, and smooth ER, which lacks ribosomes. About one-third of proteins in the cell are translated from mRNA to proteins and modified in the rough ER, where they go through posttranslational processing events including assembly, glycation, and disulfide bond formation, ultimately adopting their active protein structure [1]. The smooth ER is also the site of lipid and steroid synthesis and a calcium reservoir, with the latter function playing an important role in regulating calcium levels in the cell [2,3]. However, introduction of immature proteins to the extent that it exceeds the ER’s capacity, thereby overwhelming the ER and impairing its function, or depletion of calcium within the ER, results in a state called ER stress [3,4,5]. These events induce a variety of cellular defense mechanisms that collectively serve to promote survival in a process called the ER stress response. This ER stress response is well documented in cell types characterized by active protein secretion, such as plasma cells, pancreatic β-cells, hepatocytes, and osteoblasts. A number of recent studies have reported that the ER stress response is involved in the pathogenesis of ischemic diseases, viral infectious diseases, hyperhomocysteinemia, diabetes, neurodegenerative diseases, metabolic syndrome, and cancers [1,6,7,8,9,10,11,12,13]. Like other organs, upper respiratory tract regions encompassing the ear, nose, and throat—the domain of the otorhinolaryngology field—are susceptible to various diseases, including infectious disease, ischemic disease, and cancer. Given these shared vulnerabilities, the pathogenesis of various otorhinolaryngology-related diseases may also be closely related to the ER stress response. To the best of our knowledge, there has yet to be a systematic evaluation of the literature on the relationship between the ER stress response and various otorhinolaryngological diseases.

Accordingly, in this review we address these gaps in our knowledge by summarizing research on the relationship between ER stress response and otorhinolaryngologic diseases, focusing on the mechanisms by which ER stress affects each disease.

## 2. ER Stress Response

The ER regulates the flow of macromolecules—carbohydrates, proteins, and lipids—needed to maintain cellular function. Faced with challenges to this essential homeostatic mechanism, organelles initiate several paths towards recovery, collectively called the ER stress response [1,2,3,4,5]. Specifically, ER stress causes the cell to engage in processes designed to overcome the resulting stress; these processes are mediated by three signaling systems: pancreatic ER kinase (PERK), inositol-requiring 1α (IRE-1α)/X-box binding protein 1 (XBP-1), and activating transcription factor 6 (ATF6) [1,2,5]. The ER stress response can be further categorized into four different types.

### 2.1. Translational Attenuation of mRNA into Proteins Due to ER Stress

The first involves attenuation of translation of mRNA into proteins, which serves to reduce the introduction of new proteins into the ER [14]. Phosphorylation of eukaryotic translation initiation factor 2 alpha subunit (eIF2α) is responsible for the reduced translation of mRNA to proteins [15].

eIF2α is a member of the translation initiation complex that carries methionine to the start codon AUG. When eIF2α is phosphorylated and inactivated, the binding of methionine-tRNA, which is the initiation site of ribosome, is hindered. Then the frequency of recognition for the AUG start codon is reduced, and the translation into protein from mRNA is reduced [10]. Phosphorylation of eIF2α during ER stress occurs by the PERK. PERK is a type 1 transmembrane serine/threonine kinase situated on the ER membrane, and it stays inactive by binding with ER chaperone protein, the binding immunoglobulin protein (BiP, also known as glucose regulatory protein (GRP)-78). However, when the unfolded proteins increase within the ER lumen, BiP dissociates from PERK and binds with unfolded proteins to aid the proteins to be folded appropriately. The dissociated PERK is then oligomerized and activated by trans-autophosphorylation and phosphorylates eIF2α [12]. Eventually, PERK activation by ER stress decreases the proteins to be synthesized from mRNAs that suppress the novel proteins to enter the ER [3,4,5,12]. When eIF2α is phosphorylated due to the activation of PERK, protein synthesis is generally suppressed, but translation of ATF4 protein from mRNA is rather increased [13]. This response works as a mechanism for a cell to recover from the state of translation attenuation due to ER stress. In other words, ATF4 activates its subsequent signal transduction pathway, CHOP, along with growth arrest and DNA damage-inducible protein (GADD34), and GADD34 dephosphorylates PP1 (protein phosphatase 1) and eIF2α and forms a new translation initiation complex [5,12].

### 2.2. Transcription Activation Due to ER Stress

Second is activation of the ER-resident IRE1/XBP-1 and ATF6 signal transduction systems, which induce the expression of protein-folding ER chaperones such as Bip, improving the protein-folding function of the ER [16,17]. IRE1, like PERK, binds with BiP in an environment absent of stress, and it dissociates from BiP and autophosphorylates itself to be activated when the ER stress occurs. Activated IRE1 splices XBP-1 mRNA to enable its expression as an active XBP-1 protein [3,4,5]. XBP-1 is a transcription factor that induces transcription activation by binding to the promoter of the ER stress element (ERSE) gene, which its expression is increased in the events of ER stress [3]. IRE1/XBP-1 signal transduction pathway is especially known to play an important role in cells that actively secrete proteins such as plasma cells, hepatocytes, and exocrine pancreatic cells. ATF6 is a transcription factor that is present on the ER membrane which is transferred to the Golgi apparatus from ER, cleaved by site-1 protease (S1P) and site-2 protease (S2P). Its cleaved N-terminal ATF6 (p60) then moves into the nucleus and works directly as a transcription factor to drive expression of enzymes and chaperones that are significant for protein folding. Additionally, an active form of ATF6 can bind to the transcription promoter region of ERSE to induce the expression of XBP-1 mRNA. In the events of ER stress, ATF6 that moved into the nucleus induces the expression of XBP-1 mRNA, and the expressed XBP-1 mRNA is spliced by the RNase activation of IRE1 to generate an active form of XBP-1 protein. In short, the activation of ATF6 occurs rapidly during ER stress because the pre-existing proteins merely need to be dissociated to be activated, whereas the activation of XBP-1 occurs relatively slowly because it requires transcription and splicing of mRNA and synthesis of protein to be activated. Therefore, it can be postulated that the early ER stress response is promptly taken place by ATF6 and the later and continuous ER stress response is mediated by the activation of XBP-1 [7,8,17].

### 2.3. ER-Associated Protein Degradation

Third is ER stress-associated degradation (ERAD), a process that degrades and removes misfolded or unfolded proteins via the cell’s ubiquitin-proteasome system [18]. The first step in ERAD is to recognize a misfolded protein, the mechanism of which is not well known, but it is assumed that Man8-binding protein lecitin plays an important role in glycoproteins. In the second step, calnexin or calreticulin binds to misfolded proteins, preventing immature proteins from being transported to the Golgi apparatus. Misfolded proteins go through a refolding process at this step. In step three, misfolded proteins are carried to the cytoplasm through the Sec61p pathway. In the cytoplasm, they go through deglycosylation and polyubiquitination, and ultimately are degraded by the 26S proteasome [19,20].

### 2.4. Apoptosis

The final type of ER stress response is removal of damaged cells by apoptosis. If the ER stress is too dire—beyond the point of recovery by the above three responses—the damaged cell removes itself by activating the apoptosis pathway [3,18]. A number of different apoptosis pathways are activated during ER stress, with a typical example being apoptosis driven by the C/EBP family member, CHOP (C/EBP homologous protein) [21]. Transcription activation of CHOP gene is regulated by its upper signal cascade molecules during the ER stress response, such as PERK, ATF6, and Ire1. CHOP knockout cells are resistant to the apoptosis driven by ER stress, whereas the overexpression of CHOP promotes apoptosis [18,21]. Caspase-12, an ER-proapoptotic cysteine protease present within the ER membrane, and c-Jun N-terminal kinases (JNK) pathways also induce apoptosis in response to extreme ER stress [22,23].

## 3. ER Stress, Unfolded Protein Response, and Autophagy

The unfolded protein response (UPR) is a cellular stress response related to the ER stress. ERAD and UPR are two key quality-control machineries in the cell. ERAD is responsible for the clearance of misfolded proteins in the ER for cytosolic proteasomal degradation, while UPR is activated in response to an accumulation of unfolded or misfolded proteins in the lumen of the ER. The UPR has three aims: initially to restore normal function of the cell by halting protein translation, degrading misfolded proteins, and activating the signaling pathways that lead to increasing the production of molecular chaperones involved in protein folding. If these objectives are not achieved within a certain time span or the disruption is prolonged, the UPR aims towards apoptosis [24,25]. UPR is initiated by three ER transmembrane proteins: IRE1α, PERK, and ATF6. The common theme among these three ER stress sensors is that they all contain an ER-luminal domain believed to be capable of directly or indirectly sensing misfolded proteins when the latter reach critically high concentrations. Luminal domain sensing of misfolded proteins leads to changes in the oligomerization state of each sensor and activation of their associated downstream activities, thus transducing a signal from the ER lumen into the cytoplasm. The initial phases of UPR activation have two key roles. The first is attenuation of translation and cell cycle arrest by the PERK receptor. This occurs within minutes to hours of UPR activation to prevent further translational loading of the ER. The activated cytosolic domain causes translational attenuation by directly phosphorylating the α subunit of the regulating initiator of the mRNA translation machinery, eIF2. This also produces translational attenuation of the protein machinery involved in running the cell cycle, producing cell cycle arrest in the G1 phase [25]. The second is the increase in the production of proteins involved in the functions of the UPR. UPR activation also results in upregulation of proteins involved in chaperoning misfolding proteins, protein folding and ERAD, including further production of BiP [24]. Ultimately, this increases the cell’s molecular mechanisms by which it can deal with the misfolded protein load. The aim of these responses is to remove the accumulated protein load whilst preventing any further addition to the stress, so that normal function of the ER can be restored as soon as possible. If the UPR pathway is activated in an abnormal fashion, this activates cellular stress signaling and inflammatory pathways because of the abnormal conditions disrupting ER homeostasis [24,25].

In above mentioned, accumulation of misfolded proteins causes stress and activates the UPR to induce the expression of chaperones and proteins involved in the recovery process in ER. Eukaryotic cells have evolved strategies to respond to stress conditions. For example, autophagy in yeast is primarily a response to the stress of nutrient limitation. Autophagy primitively portrayed as an evolutionarily conserved process is involved in cellular homeostasis by facilitating the lysosomal degradation pathway for the recycling and elimination of intracellular defective macromolecules and organelles. ER stress serves dual roles by favoring mechanisms of both autophagy induction and inhibition through regulation of Ca2+, IRE1α, PERK, and the ATF6α signaling pathway. Generally, autophagy serves as an adaptive stress response that helps to sustain cell survival during physiological ER stress, whereas pathological ER stress can lead to the inhibition of autophagy [26].

## 4. ER Stress and Inflammation Responses

Beyond the adaptive phase of restoring ER homeostasis, ER stress and activation of the ER signaling pathway can affect host immune function and promote inflammation. The ER-stress observed in inflammatory pathology determines the nature and duration of the immune response [27]. The ER-stress response and inflammation are interconnected through various mechanisms. Mutations that produce defects in protein folding or in the ER-stress response pathways lead to spontaneous inflammation and hyper-inflammatory responses [27]. The production of reactive oxygen species (ROS), the activation of the transcription factor nuclear factor-kB (NF-kB) and the induction of acute-phase proteins have been linked to both ER-stress and inflammatory responses. ER stress reportedly activates NF-κB signaling, and this was considered a mechanism for inducing inflammatory responses [28,29]. ER-stress pathways are also implicated in regulating immunogenicity. ER-stress specific signal may increase immunogenicity by tagging the cell as stressed. ER stress induces inflammatory responses by activating UPR transcription factors such as XBP1s, ATF6, and cAMP response element-binding protein H (CREBH) [30]. ER stress activates inflammatory signaling cascades through the interaction between UPR components and canonical cytokine-regulatory transcription factors. Increased expression of pro-inflammatory cytokines during ER stress suggests roles of the UPR in inflammatory disorders [31].

## 5. Association of ER Stress with Otologic Diseases

### 5.1. Otitis Media

Otitis media (OM) refers to all inflammatory diseases occurring in the middle ear cavity, most of which are caused by Eustachian tube dysfunction and upper respiratory tract infection by a pathogen [32]. When inflammation takes place in the middle ear cavity, a variety of immune responses give rise to various inflammatory mediators. ER stress responses are involved in chronic inflammatory reactions caused by various infectious diseases [33,34]. These observations have prompted discussions about the possibility that ER stress affects various pathogenic ER processes related to immunity, a hypothesis specifically evaluated by two studies (Table 1). One of these studies examined six ER stress-related genes—*IRE1α*, spliced *XBP1 (sXBP1), PERK, CHOP, ATF6, and BiP*—through quantitative reverse transcription polymerase chain reaction (qRT-PCR) of inflammatory mucosal tissue taken from the middle ear cavity of 47 patients diagnosed with chronic otitis media (COM) (>3 months of morbidity) [32]. According to this study, sXBP1 mRNA expression was significantly higher in the group with otorrhea than in the group without it. ATF6 expression was significantly higher in the group with ossicular destruction compared with the ossicle-intact group. mRNA expression of the six ER stress-related genes did not differ significantly between patients with positive microbial cultures and those with negative cultures or between those with facial nerve dehiscence and those without facial nerve dehiscence. Also, because the six ER stress-related genes were expressed in all target samples, the authors suggested that this supports involvement of the ER stress reaction in the pathogenesis of COM. Among these six genes, sXBP1 was expressed at the highest levels and IRE1 at the lowest, suggesting that ERAD activity is more closely related than translational attenuation or transcriptional induction in the pathophysiological mechanism of COM. In addition, transcriptional induction of sXBP1, CHOP, and BiP mRNA was higher than that of PERK, IRE1α, and ATF6 mRNA during the early stage of ER stress, suggesting that ER stress is already active in COM. Another study that identified a relationship between ER stress and OM reported expression of the same six ER stress-related genes in exudates from the middle ear cavity of 39 pediatric OM with effusion (OME) patients [35]. In this latter study, the authors collected middle ear effusions from children in which a ventilation tube had been inserted and examined expression levels using qRT-PCR. These authors found that expression of CHOP was higher in the otitis-prone group than in the non-otitis–prone group. The most common type of fluid was mucoid, and IRE1α expression was higher in other fluid types. The authors proposed that ER stress-related responses are activated in pediatric OME patients, and that specific ER-stress related pathways are related to both the characteristics of the fluid and the frequency of OME. ATF6, IRE1, and PERK are three major transmembrane proteins involved in the ER stress response: sXBP1 and CHOP are the signaling messengers for IRE1 and PERK, respectively, and BiP is an indicator of inhibition of the ER stress response [35]. Therefore, findings from previous studies assessing expression levels of the six types of ER stress-related genes––IRE1α, sXBP1, PERK, CHOP, ATF6, and BiP––could provide evidence that the ER stress response plays various roles in the pathogenesis of OM.

### 5.2. Hearing Loss

Hearing loss (HL), affecting over 50 million people worldwide, is the most frequent and important symptom of ear disease caused by partial or complete dysfunction of the auditory pathway from the ear to the cerebral auditory cortex. As such, it is a significant public health concern. HL is not limited to hearing deficits alone, but rather includes difficulties in comprehension and understanding of speech, which can have significant effects on the quality of life. HL can be classified into conductive HL (CHL) and sensorineural HL (SNHL). CHL is mainly caused by trauma to the ear or OM, whereas SNHL is caused by various factors, including nerve damage in the auditory circuit and damage to the cochlea. In addition, various animal studies have reported that ER stress contributes to the pathology of SNHL. Table 1 summarizes reports investigating the relationship between HL and ER stress.

Herranen et al. conducted animal studies on MANF (mesencephalic astrocyte-derived neurotrophic factor)-related mechanisms underlying the development of HL in outer hair cells (OHCs) of the cochlea. In this study, MANF inactivation caused the death of OHCs, an effect that increased as the hearing threshold was increased. Moreover, all OHCs formed normally during development in MANF-inactivated mice, but progressive OHC death became evident soon after the onset of hearing function. Additionally, a MANF deficiency becomes detrimental when accompanied by gene mutations that predispose to HL, which act by intensifying ER dyshomeostasis. On the basis of these observations, the authors of this study concluded that MANF might serve as a therapeutic candidate for protection against HL induced by stressors that target the ER machinery [36].

It has been reported that ER stress induces apoptosis in auditory cells, resulting in acute SNHL. One study established an animal model of acute SNHL using the mitochondrial toxin 3-nitropropionic acid (3-NP). In this study, researchers examined the time course of CHOP expression, and apoptosis and degeneration of fibrocytes after administration of 3-NP. They found that, in cases where primary injuries were detected, expression of the ER stress markers, CHOP and ATF4, was upregulated in the cochlear lateral wall, suggesting that ER stress plays a role during the onset or exacerbation of HL in some types of auditory disorders [37]. Another study reported that tunicamycin-induced apoptosis was characterized by the expression of CHOP, a specific ER stress-associated pro-apoptotic factor, in hair cells and spinal ganglion cells in the cochlea. These researchers established a novel animal model of HL by perilymphatic perfusion of tunicamycin, an ER stress activator that inhibits N-acetylglucosamine transferases. Subacute and progressive HL was observed at all sound frequencies studied, and induction of ER stress marker genes was noted in the cochlea. Among cell types in the cochlea, OHCs were the most sensitive to ER stress. An electron microscopic analysis demonstrated degeneration of subcellular organelles in inner hair cells and nerve endings of spiral ganglion cells. Induced expression of the ER stress markers, CHOP, ATF4 and GRP94, in cochleae following treatment with tunicamycin is direct evidence for ER stress [38].

There have also been reports on the relationship between XBP1 and forkhead box O1 (FoxO1) in ER stress in tunicamycin-induced HL in the HEI-OC1 (House Ear Institute-Organ of Corti 1) cell line. XBP1 is a major regulator of the UPR that mediates the adaptation to ER stress, whereas FoxO1 is a transcription factor involved in several important biological processes, including cell-cycle arrest, apoptosis and aging. Notably, the major ER stress sensor, IRE1α, is a transmembrane RNase involved in XBP1 mRNA splicing. Based on these observations, the authors analyzed the effects of ER stress on auditory cells, focusing on XBP1, FoxO1, and autophagy function. They found that tunicamycin-induced ER stress resulted in IRE1α-mediated XBP1 mRNA splicing and autophagy, and further showed that these splicing events and FoxO1 activity were involved in ER stress-induced autophagy. Knocked down of XBP1, IRE1α or FoxO1 by siRNA in auditory cells significantly blocked the expression of microtubule associated protein 1 light chain 3-II (LC3-II). LC3-II is necessary for the process of autophagosome formation. Therefore, these results indicate that autophagy was induced in auditory cells on ER stress condition. The relationship between XBP1 and FoxO1 by small interfering RNA (siRNA) paradoxically showed negative regulation of FoxO1 expression by XBP1. Thus, the authors suggested that XBP1-FoxO1 interaction regulated the ER stress-induced autophagy in auditory cells [39].

In another study, changes in the density of spiral ganglion cells and the expression of BiP, IRE1α, AFT-6α, PERK, eukaryotic initiation factor 2α (eIF2α), CHOP, and caspase-12 were found at various times after kanamycin treatment. In this study, the number of spiral ganglion cells deleted reached ~50% on day 70 after kanamycin administration, at which time the ER of most spiral ganglion cells was dilated. CHOP, BiP, eIF2α, IRE1α, PERK, and caspase-12 expression were also significantly unregulated after kanamycin treatment. The number of spiral ganglion cells positive for both terminal deoxynucleotidyl transferase-mediated dUTP nick-end labeling (TUNEL) staining and caspase-12 (i.e., apoptotic cells) increased between 7 and 28 days. Taken together, these data demonstrate that ER stress is involved in kanamycin-induced apoptosis of spiral ganglion neurons, which is mediated, at least in part, by ER stress-induced upregulation of CHOP and caspase-12 [40].

Another study observed links between aminoglycoside-induced mistranslation and protein misfolding on the one hand, and neuropathy in XBP1-haploinsufficient (XBP1^+/−^) mice. These authors demonstrated that aminoglycosides induce misreading in mammalian cells; they also assessed ER stress and UPR pathways. Intra-tympanic aminoglycoside treatment caused high-frequency HL in XBP1^+/−^ mice, but not in wild-type littermates. Densities of spiral ganglion cells and synaptic ribbons were decreased in gentamicin-treated XBP1^+/−^ mice, whereas sensory cells were preserved. These results suggest that aminoglycoside-induced ER stress and cell death in spiral ganglion neurons is mitigated by XBP1, masking aminoglycoside neurotoxicity at the organismal level [41].

These researchers also studied the mechanism of ER stress-induced cell death in auditory cells using HEI-OC1 cells and the known ER stress inducer, tunicamycin. They demonstrated that ER stress not only initiates caspase-9/caspase-3–dependent intrinsic apoptosis, but also induces receptor-interacting serine/threonine-protein kinase 1 (RIPK1)-dependent necroptosis in auditory cells. They further showed that ER stress-induced necroptosis was dependent on the induction of RIPK1, which is negatively regulated by caspase-8 in auditory cells. These results suggest that ER stress-induced intrinsic apoptosis depends on the induction of caspase-9 and caspase-3 in auditory cells. According to this study, necroptosis could serve as a backup apoptotic cell death route in auditory cells experiencing ER stress [42].

Park et al. reported that overdoses of pyridoxine (vitamin B6)—one of the most widely used vitamin supplements or medicines—cause auditory neuropathy. Notably, they further showed that ER stress is also involved in pyridoxine-induced ototoxicity. To explore the direct mechanism underlying the effects of pyridoxine on auditory neuropathy, they performed studies on organ of Corti explants *ex vivo* and ventral otocyst-neuroblast cell line number 33 (VOT-33), a cochlear neuroblast cell line, *in vitro*. Pyridoxine induced VOT-33 apoptosis, as indicated by accumulation of a sub-G_0_/G_1_ fraction, caspase-3 activation, and poly(ADP-ribose) polymerase (PARP) cleavage. In addition, pyridoxine induced the generation of reactive oxygen species (ROS) and altered mitochondrial membrane potential transition (MPT). It also increased expression of Bcl-2 family proteins, subsequent accumulation of Ca^2+^, and changes in expression of the ER stress-related proteins, CHOP, BiP, phosphorylated PERK (p-PERK) and caspase-12. They suggested that pyridoxine preferentially induces extensive cell death in nerve fibers in primary organ of Corti explants and markedly increased apoptotic cell death in VOT-33 cells via mitochondria-mediated ER stress [43].

There have been reports relating ER stress with HL through genetic mutations. Over 100 genes are associated with HL in humans, more than 30 of which have been identified. Among identified genes associated with HL are several that encode gap junction proteins. According to a previous study, mutations in the gene encoding the gap junction protein connexin-31 (Cx31) are associated with HL. Using Cx31-mutant and wild-type (Cx31wt) mice, Xia K et al. examined whether ER stress disables gap junction proteins functionally or morphologically and whether this, in turn, causes HL. In this study, exogenously expressed Cx31wt formed functional gap junctions at cell-cell contacts. In contrast, HL-associated Cx31 mutants resided primarily in the ER and Golgi-like intracellular punctate structures and failed to mediate Lucifer yellow transfer. They further showed that expression of Cx31 mutants, but not Cx31wt, led to upregulation of the ER chaperone BiP and increased BiP–Cx31 association, indicating induction of ER stress. Thus, they suggested that HL-associated Cx31 mutants show impaired trafficking and promote ER stress, and hence lose the ability to assemble functional gap junctions [44].

A separate study explored the relationship between ER stress and transmembrane and tetratricopeptide repeat 4 (Tmtc4), which is broadly expressed in the mouse cochlea and is important for normal hearing, as evidenced by the fact that genetic inactivation of *Tmtc4* induces HL. These authors showed that Tmtc4 was enriched in the ER and functioned by regulating Ca^2+^ dynamics and the UPR. In a mouse model, small-molecule inhibitors of the UPR and ISRIB (integrated stress response inhibitor), a stress-response modulator, prevented HL by activating eIF2β. Moreover, mice with homozygous inactivation of both *Tmtc4* and *Ddit3* (encoding CHOP) had less extreme HL than mice with knockout of *Tmtc4* alone [45]. Another study of ER stress-related HL employed *Cdh23^erl/erl^* mutant mice. Cadherin 23 (Cdh23) protein is localized to the upper part of the tip link in hair cells, and in humans, *Cdh23* mutations cause nonsyndromic autosomal recessive deafness (DFNB12) and Usher syndrome type 1D. This study revealed that mutant Cdh23 was unable to fully reach the top of hair bundles and became co-localized with BiP in subapical regions of OHCs in *Cdh23^erl/erl^* mutant mice. They showed that the PERK arm of the UPR was activated in cochleae of *Cdh23^erl/erl^* mutant mice and also showed that disruption of the *Ddit3* gene protected hearing and OHCs in *Cdh23^erl/erl^* mutant mice. They further reported that the small-molecule ER stress inhibitor, salubrinal (Sal), slowed the progression of HL and hair cell death, prevented HL, and protected against OHC death in Cdh23^erl/erl^ mutant mice. Sal indirectly inhibits eIF2 as a result of reduced dephosphorylation of its α-subunit, resulting in activation of stress response pathways usually triggered by events such as oxidative stress or buildup of unfolded protein in the ER [46]. It also suppressed ER stress-induced apoptosis in *Cdh23^erl/erl^* mutant mice. On the basis of these results, the authors suggested that ER stress-induced hair cell apoptosis plays a key role in HL and hair cell death in *Cdh23^erl/erl^* mutant mice, and that protein synthesis and related protein-folding processes are early molecular events in ER stress and apoptosis [46].

Noise-induced HL (NIHL), which initially causes injury to OHCs, is a critical factor in acute SNHL in certain regions of the world, including Asia, and occurs through two types of cell death mechanisms: apoptosis and necrosis. Studies examining the involvement of ER stress in apoptosis or necrosis have identified the molecular chaperone BiP and the ER stress-specific transcription factor CHOP as major indicators of ER stress. A case-control study on the mechanism of ER stress in NIHL using guinea pigs as a model showed that noise exposure caused apoptosis, necrosis and loss of OHCs, and that apoptotic cells were increased to a greater extent than necrotic cells on post-exposure days 1 and 4. BiP levels were also significantly higher in all three experimental groups compared with the control group. CHOP levels were increased 1 day after exposure, reaching a peak that was maintained until 4 days, after which it returned to baseline levels by 14 days post-exposure. On the basis of these results, the authors speculated that the ER stress response was activated by induction of BiP expression, which served to lessen the extent of the resulting cellular damage, and by activation of the CHOP pathway, which eliminated the most severely damaged cells [47].

Another study investigated age-related HL (ARHL), another type of SNHL, by analyzing the expression of ER stress-related factors as a function of age in C57BL/6 mice, a representative animal model of ARHL. In this study, the reduced expression of BiP and increased numbers of ubiquitinated proteins observed in cochleae of aged mice suggested that the capacity of the UPR was impaired and that the cell death pathway was activated. They found a marked increase in expression of the ER-related pro-apoptotic factor CHOP in cochleae of aged mice, whereas the level of cleaved caspase-12 did not differ between the two groups. In addition, cleaved caspase-9, caspase-3 and PARP1 were significantly increased in aged cochleae, suggesting that activation of apoptosis in the cochlea results from crosstalk between the ER and mitochondria through CHOP. These results indicate that impaired UPR in cochleae of aged C57BL/6 mice and attendant ER stress may lead to apoptosis via the mitochondrial pathway and that ER stress-induced apoptosis may not be mediated by caspase-12 [48].

Aminoglycosides such as kanamycin and tunicamycin exert well-known ototoxic effects, as noted above. However, there are reports on the role of ER stress in ototoxicity associated with other HL-inducing drugs. One such drug is N-acetyl-para-aminophenol (APAP), better known as acetaminophen, which is most commonly used as a pain reliever. Although commonly used, acetaminophen is reported to cause ototoxicity through an ER stress response as a side effect when the drug is taken long term. APAP is a pro-drug metabolized by the cytochrome P450 2E1 isozyme (CYP2E1) to an electrophilic metabolite called N-acetyl-p-benzoquinone imine (NAPQI). Using the HEI-OC1 cell line, these authors sought to determine whether ROS and ER stress contribute to the mechanism by which APAP and its metabolite, NAPQI, exert cytotoxic effects in auditory cells. They found that both APAP and NAPQI induce expression of the ER stress markers, BiP and CHOP. iTRAQ gene and protein expression datasets indicated that APAP- and NAPQI-induced death of HEI-OC1 cells could be mediated, at least in part, by ER stress and functionally related alterations in protein folding and degradation. The cytotoxic effects of APAP and NAPQI on HEI-OC1 cells are dependent on eIF2a and CHOP, but independent of IRE1-, ATF6-, and ATF4-mediated signaling. Thus, these authors suggested that ER stress is a shared mechanism underlying the toxic effects of APAP and NAPQI on HEI-OC1 cells [49].

Another study examined the correlation between HL and ER stress in rats treated with cisplatin, an anticancer drug that exhibits dose-dependent ototoxicity. This study revealed that treatment with cisplatin increased the levels of active caspase-12 in cochlear cells, a finding indicative of cisplatin-induced activation of ER-specific apoptosis. The increased expression of CHOP and cleaved caspase-9 suggested a close relationship between severe ER stress and mitochondria-dependent apoptosis in cochlear cells of cisplatin-treated rats [50].

One recent study reported the effect of ER stress on reversible HL in bilirubin-induced HL through animal models using mice. The *in vitro* results indicated that bilirubin induces changes in gene expression consistent with ER stress and activation of the UPR. It also induced gene expression changes associated with inflammation and NF-κB activation. The in vivo model showed a raised auditory threshold. Whole genome gene expression analysis confirmed inflammation as a key mechanism of bilirubin neurotoxicity in the auditory pathway and shared gene expression hallmarks induced by exposure to bacterial lipopolysaccharide (LPS) a well-characterized inducer of neuroinflammation. Interestingly, bilirubin caused more severe damage to the auditory system than LPS in this model, the HL was protected by perturbing the inflammatory response. Also, anti-inflammatory compounds (interfering with NF-κB and TNFα signaling) protected the auditory system from bilirubin toxicity. In summary, bilirubin increased the hearing threshold through mechanisms such as increased ER stress, induction of inflammatory responses, and activation of the NF-kB pathway [51].

Collectively these observations suggest that, in most cases, HL manifests when OHCs in the organ of Corti are damaged or rendered dysfunctional by ER stress. They further imply that factors that change the normal physiological functioning of OHCs, even when these factors are as varied as drugs, aging, exposure to noise or genetic mutation, promote ER stress. Overcoming these factors may require the cell to activate different series of ER stress responses to varying degrees, and possibly apoptosis, to elicit HL. Therefore, elucidating the mechanism underlying the ER stress response would provide a new therapeutic target for HL.

## 6. Association of ER Stress with Rhinologic Diseases

### 6.1. Allergic Rhinitis

Type 2 allergic immune responses are typically characterized by eosinophilic inflammation accompanied by increases in type 2 cytokines such as interleukin (IL)-4, IL-5, and IL-13 in affected tissues and blood. These responses are closely associated with various allergic diseases, including allergic rhinitis (AR), a hallmark of which is the presence of serum antigen-specific immunoglobulin E (IgE) indicative of adaptive immunity involving type 2 helper T cells (T_H_2 cells) [52].

The allergic response begins at the interface between the external environment and the epithelium and subsequently involves diverse cell types at all levels of innate and adaptive immunity. During these processes, cells produce large amounts of secretory proteins to defend themselves against endogenous and exogenous threats and/or to efficiently communicate with other cell types to generate an organized immune response. Thus, proper functioning of the ER and maintenance of protein homeostasis is critical for these cells. In these processes, the influence of ER stress is not restricted to protein folding, but instead can intersect with immunity at many levels, thereby leading to complex chronic inflammatory diseases such as allergy. Before T cells involved in various allergic disease endotypes can be activated, the allergen must be recognized by antigen-presenting dendritic cells (DCs) and presented to T cells in draining lymph nodes. Epithelial cells are known to be key modulators in controlling DC activation through release of cytokines and endogenous associated molecules. Given the role of epithelial cells as the first line of defense, diverse coexistent environmental insults may also converge on epithelial-DC interactions [53]. For example, inflamed airway epithelial cells exhibit overt signs of ER stress, and bronchial epithelial XBP-1 has been reported to mediate inflammation-induced ER/Ca^2+^ store expansion, which amplifies Ca^2+^-dependent secretion of cytokines [54,55]. Thus, ER stress and the UPR pathway may be central players in the regulation of epithelial-DC interactions, which are crucial for the initiation and amplification of allergic response in the respiratory tract. In addition, XBP1 plays a pivotal role in the terminal differentiation of B cells into highly secretory plasma cells by mediating expansion of the ER and synthesis of proteins required for antibody production and secretion [56]. Moreover, the IRE1α-XBP1 arm of the UPR is thought to be important in the early developmental stage of B cells and terminal differentiation of effector CD8^+^ T cells [55,57]. Notably, considering that XBP-1 mRNA in naïve B cells is uniquely induced by IL-4, this arm of the UPR may be particularly important in the type-2 allergic response [58]. Additionally, differentiation of eosinophils—a subset of granulocytes—from myeloid cell progenitors is uniquely dependent on the IRE1α-XBP1 pathway, and deletion of XBP1 results in massive defects in eosinophil maturation [59]. Moreover, macrophages are known to connect cell surface innate toll-like receptor (TLR) signaling with intracellular IRE1α-XBP1 pathway-mediated secretion of pro-inflammatory cytokines, thereby linking the ER and UPR pathway to innate effector function in macrophages [60]. Concurrent with this, TLR signaling suppresses the ATF4-CHOP-mediated cellular apoptotic pathway to effectively coordinate the innate function of macrophages [61]. Table 2 summarizes reports investigating the relationship between AR and ER stress. A recent in vitro study expanded on these observations in a primary cultured human nasal epithelial cell model of allergic rhinitis caused by the house dust mite (HDM). These researchers used HDM as an allergen at a concentration sufficient to induce an allergic reaction in primary cultured human nasal epithelial cells isolated from two non-asthmatic subjects. Cells from both subjects exhibited increases in p-IRE, as well as increases in the ER chaperone BiP, GRP94, and ER protein 57 (ERp57) 72 hours after exposure to HDM. Expression of the ER stress transducer ATF6α and the downstream transcriptional effector CHOP were also increased after HDM exposure. Allergen exposure was accompanied by activation of caspase-3 to varying degrees in primary human nasal epithelial cells. The authors of this study suggested that HDM induces ER stress in airway epithelial cells and that ATF6α and ERp57 play a significant role in the development of cardinal features of allergic airways disease, such as AR [62].

As previously mentioned, a significant portion of allergic disease, such as AR, is associated with the T_H_2 immune response. However, in a recent study, only 50% of patients with asthma were associated with a type 2 immune response. Therefore, research on non-type 2 immune response has been actively conducted recently [72]. Generally, both type 17 (T_H_17) and type 1 (T_H_1) responses are often associated with neutrophilic inflammation of airways. In recent study, corticosteroid-insensitive severe asthma possesses mixed type 17/type 1 immune response in the background of variable type 2 immunity [72]. In other study, IFN-γ has been implicated in bronchial asthma pathogenesis through T_H_2-independent IFN-γ/mast cell axis as well as its classical effects on T_H_2 cells [73]. In addition, LPS-induced ER stress leads to the increased expression of IL-17 in airway epithelium, thereby further potentiating ER stress and NF-κB activation via forming a positive feedback loop in airway epithelial cells [74,75]. These data suggest that ER stress and UPR pathways may play a role in non-type 2 neutrophilic allergic response. In summary, the role of ER stress in the development of AR is related to Type 2 allergic immune responses, similar to that in asthma, and some are also related to non-Th2 immune responses.

### 6.2. Chronic Rhinosinusitis

Chronic rhinosinusitis (CRS) is a very complex inflammatory disease that occurs in the nasal cavity and paranasal sinuses. It is traditionally categorized according to the presence of nasal polyp into CRS with nasal polyp (CRSwNP) and CRS without nasal polyp (CRScNP). Various pathological mechanisms are known to contribute to the incidence and aggravation of CRS. Table 2 summarizes reports investigating the relationship between CRS and ER stress. Studies have shown that *Staphylococcus aureus* exotoxins (SEs; SEA, SEC1-C3, SED) and *S. aureus* enterotoxin B (SEB) act as superantigens that affect the promotion of CRS. One study reported significantly more SEB-positive cells and higher production of ROS in the epithelial layer of eosinophilic polyps (EPs) compared with non-eosinophilic polyps (NEPs) or control tissue. The induction of BiP and p47phox (also called neutrophil cytosol factor 1 [NCF1]) was also increased significantly in EPs compared with NEPs or control mucosa. These authors also strongly detected SEB in tissues from patients with CRSwNP, and further showed that SEB induced ER stress responses in RPMI 2650 cells, a human nasal epithelial cell line. In RPMI 2650 cells, BiP elevation by SEB was reduced by pretreatment with a ROS scavenger. The authors suggested that SEB may induce ER stress via ROS production in CRSwNP, and that SEB-induced ER stress may play an important role in the pathogenesis of nasal polyposis [63].

Another study reported that 4-phenylbutylic acid (4-PBA), a chemical chaperone of ER stress, acts via the c-Src pathway to inhibit tumor growth factor-β1 (TGF-β1)-induced epithelial-mesenchymal transition (EMT) in the upper respiratory tract tissue. EMT, a biological process in which a polarized epithelial cell is transformed into a cell with a mesenchymal phenotype, characterized by enhanced mobility and invasiveness and increased production of extracellular matrix (ECM) components. The proto-oncogene product Src, a non-receptor tyrosine kinase that phosphorylates tyrosine residues on proteins, has been proposed as a possible target of the UPR in the induction of EMT. TGF-β1 is a multifunctional peptide that causes EMT in the airway epithelium and nasal tissues. It is known that EMT is causally linked to CRS and disease recalcitrance, and the role of ER stress and c-Src in TGF-β1–induced EMT has been investigated based on this relationship. The authors of this investigation reported that expression of the EMT markers E-cadherin, vimentin, fibronectin, and α-SMA was increased in NPs compared with that in inferior turbinates. TGF-β1 increased the expression of EMT markers and ER stress markers (XBP-1s and GRP78), an effect that was blocked by treatment with 4-PBA or PP2 (also known as Src and RIP2 kinase inhibitor) in A549 cells, primary nasal epithelial cells (PNECs), and organ cultures of the inferior turbinate. Also, 4-PBA and PP2 were also shown to block the effect of TGF-β1 on migration of A549 cells and suppress TGF-β1–induced expression of EMT markers in PNECs and organ cultures of the inferior turbinate. These results suggest an important role for ER stress and diverse roles for TGF-β1 in CRS [64]. In summary, ER stress may be involved in various pathways that cause chronic inflammation in CRS.

### 6.3. Intermittent Hypoxia and Obstructive Sleep Apnea

Obstructive sleep apnea (OSA) is a disorder in which all or a part of the upper respiratory tract, such as the nasal-oral-pharyngeal-laryngeal region, is partially or completely closed during sleep, resulting in repetitive hypopnea or apnea. This repetitive obstruction of the respiratory tract results in decreased oxygen levels in the blood, frequent sleep fragmentation and various additional complications, including cardiovascular impairments and dementia. In particular, intermittent hypoxia (IH), which occurs repetitively as part of this process, is closely associated with ER stress, a relationship that has been the subject of study. One such study investigated the preventive effect of adiponectin (Ad)-mediated suppression of ER stress on chronic IH (CIH) injury in the rat myocardium. Ad, a circulating cytokine derived from white adipose tissue and cardiomyocytes, has been suggested to exert cardioprotective effects by virtue of its anti-inflammatory, anti-atherogenic, anti-hypertensive, and insulin-sensitizing properties. It has also been reported that Ad levels in plasma are decreased in OSA patients compared with healthy controls. Hence, the authors of this study investigated whether ER stress is mechanistically related to the occurrence of repetitive CIH and whether Ad exerts protective effects on cardiac function in rats. They found that left ventricular function (LVF) was improved in CIH model rats after addition of Ad (CIH + Ad group). ER stress was shown to act through CHOP and BiP, which were significantly increased in the CIH-only group, to affect this process. IRE1, XBP-1, PERK, eIF2α, and ATF6 were also increased in the CIH-only group compared with normal controls—increases that were mitigated by addition of Ad. It was further shown that the percentage of apoptotic cells and the prevalence of cleaved caspase-3, -9, and -12 were significantly higher in the CIH-only group than in normal control CIH + Ad groups. Taken together, these observations suggest that cardiac function is depressed by ER stress-induced apoptosis and protected by Ad-mediated inhibition of ER stress [65]. Moreover, another study reported that DL-propargylglycine (PAG), a known effective inhibitor of cystathionine γ-lyase (CSE)-synthesized hydrogen sulfide (H_2_S), protects against myocardial injury in the context of ER stress-induced cardiac dysfunction in the CIH state in rats. Endogenous H_2_S plays an important role in maintaining cardiovascular functions, and exogenous H_2_S exerts protective effects against myocardial injury induced by various cardiovascular diseases. Predictably, inhibiting the generation of endogenous H_2_S has opposite effects. Among the findings of this study was that pretreatment with PAG significantly improved cardiac function in CIH model rats (CIH + PAG group) compared with the CIH-only group. Moreover, levels of oxidative stress, ER stress and apoptosis were significantly lower in the CIH + PAG group compared with the CIH-only group [66]. In another study in rats that addressed cardiovascular morbidity, protein expression of the proapoptotic ER stress markers, BiP, p-PERK, ATF4 and CHOP, were increased in CIH. Increases in cleaved caspase-3, an apoptosis marker, have also been shown to cause myocardial apoptosis—an IH-associated proapoptotic alteration that correlates with infarct size. In addition, application of high-intensity training in rats was reported to reduce IH-associated proapoptotic ER stress and infarct size [67]. Similarly, ER stress markers and hypoxia inducible factor-1α (HIF-1α) were increased in CIH mice, effects that were also correlated with increased infarct size. HIF-1α is known to be a factor involved in the life or death of cardiomyocites, similar to ER stress, and IH has been reported to not increase the size of infarction in mice genetically deficient in HIF-1α. Additionally, administration of tauroursodeoxycholic acid (TUDCA), a known inhibitor of ER stress, suppressed the enlargement of infarct size in these mice [68]. Taken together, these observations suggest that ER stress is a significant detrimental factor in the essential pathology of patients with OSA, the cardiovascular morbidity associated with CIH. Therefore, if the ER stress response is properly regulated in OSA patients or the factors that suppress ER stress are appropriately managed, the prediction is that cardiovascular morbidity will be decreased.

TUDCA, which is known to suppress ER stress, is also reported to prevent liver damage in mice exposed to CIH. In particular, TUDCA inhibited the dissociation between GRP78 and PERK, resulting in reduced ER stress-mediated cell death [69]. Specifically, administration of TUDCA attenuated pathological changes in the liver, reduced serum alanine aminotransferase and aspartate aminotransferase levels, suppressed ROS activity, decreased TNF-α and IL-1β levels, and inhibited hepatocyte apoptosis induced by CIH. TUDCA also inhibited CIH-induced ER stress in the liver, as evidenced by decreased expression of BiP, UPR transducers, and ER proapoptotic proteins. Taken together, the results of this latter study describe a liver-protective effect of TUDCA in CIH model mice, an effect that is mediated, at least in part, by inhibition of ER stress [69].

Another similar animal study reported the mechanism of ER stress in CIH-induced brain damage in rats. In this study, overall cognitive function was decreased in CIH model rats, and BiP expression was increased in rats with hippocampal and prefrontal lesions. These data indicate that CIH induces ER stress in the hippocampus and prefrontal cortex. CIH also induced a time-dependent, progressive increase in p-PERK levels in the hippocampus and prefrontal cortex, and downregulated p-eIF2α, a downstream target of PERK, indicating that the PERK signaling pathway is sensitive to CIH-induced ER stress in the hippocampus and prefrontal cortex. The authors of this study further found that, following CIH, p-IRE1 levels were increased, but there were no increases in spliced XBP-1 or EDEM (ER-associated degradation enhancing α-mannosidase-like protein), a downstream target of XBP-1. In addition, they demonstrated that XBP-1 mRNA splicing, activated via the IRE-1 pathway, was not significantly increased after CIH. However, they did find that ATF4 and CHOP were significantly upregulated over time after CIH, and that levels of p-JNK/JNK, apoptotic signaling kinase 1 (ASK1), and TNF receptor-associated factor 2 (TRAF2) were significantly increased. Furthermore, Sal induced a significant increase in eIF2α phosphorylation, but significantly downregulated CHOP after CIH (CIH + Sal group) compared with the CIH-only group. Sal also significantly reduced the number of apoptotic cells after CIH injury, as revealed by TUNEL staining. Collectively, these observations indicate that Sal inhibits CIH-induced neuronal apoptosis in the hippocampus and prefrontal cortex [70].

A final additional study analyzed the correlation between ER stress and the therapeutic effects of continuous positive airway pressure (CPAP) and weight loss, which are typical treatment methods for obese OSA patients. In this study, obese patients were categorized into three groups, as follows: (1) those who showed good adherence to weight loss and CPAP treatment; (2) those who showed poor adherence to treatments, and (3) those who were obese but did not have OSA. The good-adherence group showed significantly decreased expression of ATF4, CHOP, and endoplasmic reticulum oxidoreductin-1 (ERO-1) mRNAs in their adipose tissue compared with the other groups. On the basis of these results, the authors suggested that effective CPAP therapy improves obesity-associated ER stress markers in obese patients with OSA [71].

Taken together, these observations suggest that CIH—the main pathological mechanism of OSA—induces ER stress that impacts the heart, brain, and liver to increase OSA-related morbidity. Given that the known ER stress inhibitors Sal and TUDCA effectively suppressed the activation process or incidence of ER stress, the use of such agents to manage the process appropriately holds promise as a new therapeutic approach to OSA.

## 7. Conclusions

We have summarized the current knowledge regarding the association between ER stress and otorhinolaryngologic diseases (excluding cancers). Various otorhinolaryngological disorders, especially OM, HL, AR, CRS and OSA, show a clear association with ER stress. Although differences exist with respect to disease type, available evidence suggests that ER stress is involved in the pathophysiology in each case. Therefore, targeting the pathophysiological mechanisms in diseases such as OM, HL, AR, CRS, or OSA by appropriately managing ER stress could be utilized as new therapeutic strategy.

## Figures and Tables

**Table 1:** Studies assessing the association between otologic diseases and ER stress.

	Associated Diseases	Study Design	Species and/or Tissue Type	Detection Method	Target Gene(s) or Pathway(s) Associated with ER Stress	Results/Conclusion
**Dong SH, et al. [32]**	COM	Cross-sectional study	Human: inflammatory mucosal tissue in the middle ear cavity	qRT-PCR	IRE1α, sXBP1, PERK, CHOP, ATF6, BiP	sXBP1 appears to be involved in COM-associated inflammation, including otorrhea. ATF6 is associated with the destruction of ossicles.
**Kang DW, et al. [35]**	OME	Cross-sectional study	Human: effusion in the middle ear cavity	qRT-PCR	IRE1α, sXBP1, PERK, CHOP, ATF6, BiP	Expression of CHOP was higher in the otitis-prone group than in the non-otitis-prone group. The most common type of fluid was mucoid, and IRE1α expression was higher in other fluid types.
**Herranen A, et al. [36]**	HL	Animal model study	Mice: OHCs in the cochlea	Histochemistry, immunostaining, cytocochleogram	MANF	MANF inactivation resulted in the death of only OHCs. This robust OHC loss was accompanied by strongly elevated hearing thresholds. A MANF deficiency became detrimental when accompanied by gene mutations that predispose to HL through intensification of ER dyshomeostasis.
**Fujinami Y, et al. [37]**	HL (3-NP–induced)	Animal model study	Rat	qRT-PCR, immunohistochemistry	CHOP, ATF4	In a 3-NP animal model of acute HL, expression of the ER stress marker genes CHOP and ATF4 were upregulated in the cochlear lateral wall in cases where primary injuries were detected, suggesting that ER stress plays a role during the onset or exacerbation of HL in some types of auditory disorders.
**Fujinami Y, et al. [38]**	HL (tunicamycin-induced)	Animal model study	Rat	Light microscopy, fluorescence microscopy, qRT-PCR, TEM	CHOP, ATF4, BiP, GRP94, GAPDH	In the tunicamycin-induced HL model, subacute and progressive HL was observed at all sound frequencies studied, and induction of ER stress marker genes was noted in the cochlea. Among cells in the cochlea, OHCs were the most sensitive to ER stress. This study also showed degeneration of subcellular organelles of inner hair cells and nerve endings of spiral ganglion cells.
**Kishino, A, et al. [39]**	HL (tunicamycin-induced)	*In vitro* cell line study	HEI-OC1 cell line	Fluorescence microscopy, flow cytometry, Western blotting, co-immunoprecipitation, TEM, RT-PCR	IRE1α, XBP1, FoxO1, LC3-II	Tunicamycin-induced ER stress resulted in IRE1α-mediated XBP1 mRNA splicing and autophagy. XBP1 mRNA splicing and FoxO1 were found to be involved in ER stress-induced autophagy. This inference was based on the observation that expression of LC3-II was suppressed by knockdown of IRE1α, XBP1, or FoxO1. The relationship between XBP1 and FoxO1 revealed by siRNA-mediated knockdown showed a paradoxical negative regulation of FoxO1 expression by XBP1.
**Tu Y, et al. [40]**	HL (kanamycin-induced)	Animal model study	Rats	TEM, immunohistochemistry, Western blotting	BiP, IRE1α, ATF6α, p-PERK, p-eIF2α, CHOP, caspase-12	ER stress contributed to kanamycin-induced apoptosis of spiral ganglion neurons. Kanamycin treatment-induced apoptosis of spiral ganglion neurons was mediated, at least in part, by ER stress-induced upregulation of caspase-12 and CHOP.
**Oishi N, et al. [41]**	HL (aminoglycoside-induced)	Animal model study	XBP1-haploinsufficient (XBP1^+/−^) mice	Microarray analysis, proteome analysis, histochemistry, immunofluorescence assay, RT-PCR, Western blotting	XBP1	Intra-tympanic aminoglycoside treatment caused high-frequency HL in XBP1^+/−^ mice, but not in wild-type littermates. Densities of spiral ganglion cells and synaptic ribbons were decreased in gentamicin-treated XBP1^+/−^ mice, whereas sensory cells were preserved. These results suggest that aminoglycoside-induced ER stress and cell death in spiral ganglion neurons are mitigated by XBP1, masking aminoglycoside neurotoxicity at the organismal level.
**Kishino a, et al. [42]**	HL (tunicamycin-induced)	*In vitro* cell line study	HEI-OC1 cell line	Western blotting, co-immunoprecipitation, TEM, flow cytometry	Caspase-3, caspase-8, caspase-9	ER stress not only initiated caspase-9/caspase-3–dependent intrinsic apoptosis, it also induced RIPK1-dependent necroptosis in auditory cells. ER stress-induced necroptosis was dependent on the induction of RIPK1 in auditory cells, which was negatively regulated by caspase-8. Thus, caspase-8 regulates ER stress-induced necroptosis independent of the apoptosis pathway in auditory cells.
**Park C, et al. [43]**	HL (pyridoxine-induced)	*Ex vivo* animal and *in vitro* cell line study	Rat (organ of Corti explants), VOT-33 cell line	Fluorescence microscopy, immunostaining, flow cytometry, Western blotting	PERK, ATF4, BiP, CHOP, caspase-12	Pyridoxine induced VOT-33 apoptosis, as indicated by accumulation of a sub-G_0_/G_1_ fraction, caspase-3 activation, and PARP cleavage. In addition, pyridoxine induced ROS generation and alteration of MPT, including expression of Bcl-2 family protein and consequent accumulation of Ca^2+^ and changes in expression of the ER stress-related proteins, PERK, caspase-12, BiP, and CHOP.
**Xia K, et al. [44]**	HL	Animal model study	Mouse (Cx31wt vs. Cx31 mutant)	Immunofluorescence, immunostaining, immunoblotting	BiP	Exogenously expressed Cx31wt forms functional gap junction at cell-cell contacts. In contrast, HL-associated Cx31 mutants resided primarily in the ER and intracellular Golgi-like punctate structures and failed to mediate Lucifer yellow transfer. Expression of Cx31 mutants, but not Cx31wt, led to upregulation of the ER chaperone BiP and increased Cx31–BiP association, indicating induction of ER stress.
**Li J, et al. [45]**	HL	Animal model study	Mice	qRT-PCR, immunofluorescence, flow cytometry, Western blotting	CHOP, s-XBP1, BiP, caspase-3, Tmtc4	Inactivation of the gene *Tmtc4*, which is broadly expressed in the mouse cochlea, caused acquired HL in mice. TMTC4 was enriched in the ER and functioned by regulating Ca^2+^ dynamics and the UPR. ISRIB, a small-molecule modulator of the UPR and stress response that activates eIF2, prevented HL in a mouse model. Moreover, mice with homozygous inactivation of both *Tmtc4* and *Ddit3* (encoding CHOP) caused less HL than knockout of *Tmtc4* alone.
**Hu J, et al. [46]**	HL	Animal model study	Mice (*Cdh23*^erl/erl^ mutant mice vs. control B6 mice)	RT-PCR, Western blotting, immunofluorescence staining, SEM	BiP, CHOP, p-eIF2α, caspase-3	Cdh23 was unable to fully reach the top of hair bundles and became co-localized with BiP in subapical regions of OHCs in *Cdh23*^erl/erl^ mutant mice. The PERK arm of the UPR was activated in the cochleae of *Cdh23*^erl/erl^ mutant mice. Disruption of the gene encoding CHOP (*Ddit3*) protected OHCs and preserved hearing in *Cdh23*^erl/erl^ mutant mice. Sal prevented HL and protected against OHC death in *Cdh23*^erl/erl^ mutant mice.
**Xue Q, et al. [47]**	HL (noise-induced)	Animal model study (case-control)	Guinea pig	Immunohistochemistry, Western blotting, fluorescence microscopy	BiP, CHOP	BiP levels were significantly higher in all three experimental groups compared with the control group. CHOP levels were increased 1 day after exposure, reaching a peak that was maintained until 4 days before returning to baseline levels by 14 days post-exposure.
**Wang W, et al. [48]**	HL (age-related)	Animal model study	Mice (C57BL/6 mouse model of presbycusis)	Immunofluorescence staining, Western blotting	BiP, CHOP, caspase-12, caspase-3, caspase-8, PARP-1	BiP expression was reduced and the number of ubiquitinated proteins was increased in cochleae of aged mice. In aged mice, expression of the ER-related pro-apoptotic factor CHOP was markedly increased. Caspase-9, caspase-3 and PARP1 cleavage were significantly increased in aged cochleae.
**Kalinec GM, et al. [49]**	HL (APAP-induced)	*In vitro* cell line study (case-control)	HEI-OC1 cell line	Western blotting, RT-PCR, immunofluorescence, TEM, genomic and proteomic studies	CHOP, BiP, eIF2α, p-eIF2α, ATF4, XBP1	APAP induced expression of the ER stress markers, CHOP and BiP, in the HEI-OC1 cell line. The cytotoxic effect of APAP on HEI-OC1 cells was dependent on eIF2a and CHOP, but independent of IRE1-, ATF6-, and ATF4-mediated signaling.
**Zong S, et al. [50]**	HL (cisplatin-induced)	Animal model study	Rat	Immunofluorescence, Western blotting	CHOP, BiP, caspase-3, caspase-9, caspase-12	Expression of active caspase-12 was upregulated in cochlear cells of cisplatin-treated rats, indicative of cisplatin-induced activation of ER-specific apoptosis. Increased expression of CHOP and cleaved caspase-9 suggested a close relationship between severe ER stress and mitochondria-dependent apoptosis in cochlear cells of cisplatin-treated rats.
**Schiavon E, et al. [51]**	HL(bilirubin-induiced)	Animal model study	Mice	Immunohistochemistry, immuno blotting, whole genome gene expression analysis	BiP, PERK, IRE1	Bilirubin increased the hearing threshold through mechanisms such as increased ER stress, induction of inflammatory responses, and activation of the NF-kB pathway.

ER, endoplasmic reticulum; COM, chronic otitis media; OME, otitis media with effusion; RT-PCR, reverse transcription polymerase chain reaction; IRE1α, inositol-requiring enzyme 1α; XBP1, X-box-binding protein 1; PERK, endoplasmic reticulum kinase; CHOP, C/EBP-homologous; ATF, activating transcription factor; BiP, immunoglobulin heavy chain-binding protein; HL, hearing loss; OHC, outer hair cell; MANF, mesencephalic astrocyte-derived neurotrophic factor; 3-NP, mitochondrial toxin 3-nitropropionic acid; qRT-PCR, quantitative RT-PCR; TEM, transmission electron microscopy; GRP, glucose-regulated protein; GAPDH, glyceraldehyde-3-phosphate dehydrogenase; HEI-OC1, House Ear Institute-Organ of Corti 1; FoxO1, forkhead box O1; LC3-II, microtubule associated protein 1 light chain 3-II; siRNA, small interfering RNA; eIF, eukaryotic initiation factor; RIPK1, receptor-interacting serine/threonine kinase 1; VOT-N33, ventral otocyst-neuroblast cell line number 33; MPT, mitochondrial membrane potential transition; Bcl-2, B-cell lymphoma 2; Cx31, connexin-31; WT, wild type; Tmtc4, transmembrane and tetratricopeptide repeat 4; UPR, unfolded protein response; ISIRB, integrated stress response inhibitor; erl, erlong; SEM, scanning electron microscope; Sal, salubrinal; PARP, polyp(ADP-ribose) polymerase; APAP, N-acetyl-para-aminophenol (aka acetaminophen).

**Table 2:** Studies assessing the association between rhinologic diseases and ER stress.

	Associated Diseases	Study Design	Species and/or Tissue Type	Detection Method	Target Gene(s) or Pathway(s) Associated with ER Stress	Results/Conclusions
**Sidra M Hoffman, et al. [62]**	AR (induced by HDM)	In vitro cells study	Cultured primary human nasal epithelial cells	Western blotting, luminescence assay	p-IRE, GRP94, BiP, ERp57, ATF6, CHOP, caspase-3	Seventy-two hours after exposure to HDM, cell derived from subjects exhibited increases in p-IRE as well as increases in the ER chaperone BiP, GRP94, and ERp57. Expression of the ER stress transducer ATF6α and downstream transcriptional effector CHOP was also increased after HDM exposure. Allergen exposure significantly activated caspase-3 to varying degrees in primary human nasal epithelial cells.
**Kim YM, et al. [63]**	CRSwNP	In vivo (case-control) and in vitro cell line study	Human nasal epithelial cell line	Immunohistochemical staining, Western blotting	BiP, p-eIF2α, IRE1α	SEB-positive cells were more frequent, and production of ROS was greater in the epithelial layer of EPs than in NEPs or control tissue. SEB was strongly detected in tissues from patients with CRSwNP. Induction of BiP and p47^phox^ was significantly increased in EPs compared with NEPs or control mucosa. In RPMI 2650 cells, SEB-induced BiP was reduced by pretreatment with a ROS scavenger.
**Lee HM, et al. [64]**	CRS	In vitro study	Human (NP, IT)/A549 cell lines, PNECs	RT-PCR, immunofluorescence, Western blotting	XBP-1, BiP	TGF-β1 increased the expression of EMT markers (E-cadherin, fibronectin, vimentin, and α-SMA) and ER stress markers (XBP-1s and BiP), an effect that was blocked by 4-PBA or PP2 treatment. 4-PBA and PP2 also blocked the effect of TGF-β1 on migration of A549 cells and suppressed TGF-β1–induced expression of EMT markers in PNECs and organ cultures of the inferior turbinate.
**Ding W, et al. [65]**	OSA (CIH)	Animal model study (case-control)	Rat	RT-PCR, Western blotting	BiP, CHOP, p-IRE1, XBP-1, pro-ATF6, PERK, eIF2α, caspase-3, caspase-9, caspase-12	Addition of Ad increased LVF in CIH model rats (CIH + Ad group) compared with the CIH-only group. The percentage of apoptotic cells and levels of cleaved caspase-3, -9, and -12 was significantly higher in the CIH-only group compared with normal control and CIH + Ad groups. Protein Expression of cleaved caspase-3, caspase-9, and caspase-12 proteins validated TUNEL results.
**Zhou X, et al. [66]**	OSA (CIH)	Animal model study (case-control)	Rat	Western blotting	Bax, caspase-3, cleaved caspase-12, ATF6, IRE1, BiP, CHOP	Significantly lower levels of oxidative stress, apoptosis, and ER stress were detected in the CIH + PAG group compared with the CIH-only group.
**Bourdier G, et al. [67]**	OSA (CIH)	Animal model study (case-control)	Rat	Western blotting	p-eIF2α, eIF2α, ATF4, cleaved caspase-3, ATF6, p-PERK, PERK, CHOP, BiP	CIH induced cardiac proapoptotic ER stress, characterized by increased expression of BiP, p-PERK, ATF4, and CHOP. CIH-induced myocardial apoptosis was confirmed by increased expression of cleaved caspase-3. These CIH-associated proapoptotic alterations were associated with a significant increase in infarcts. HIT prevented both CIH-induced proapoptotic ER stress and increased myocardial infarct size.
**Belaidi E, et al. [68]**	OSA (CIH)	Animal model study (case-control)	Mice	RT-PCR, Western blotting	BiP, p-eIF2α, eIF2α, p-PERK, PERK, ATF4, CHOP, cleaved caspase-3, ATF6, HIF-1α	CIH induced an increase in ER-Ca^2+^ content, ER stress markers and HIF-1α activity in mice, accompanied by an enhanced infarct size. CIH failed to increase infarct size in HIF-1α–deficient mice. TUDCA totally abolished the IH-induced increase in HIF-1α activity and infarct size.
**Hou Y, et al. [69]**	OSA (CIH)	Animal model study (case-control)	Mice	ELISA, Western blotting	Cleaved caspase-3, cleaved caspase-9, BiP, PERK, ATF6, IRE1, CHOP, cleaved caspase-12, eIF2α, JNK	TUDCA inhibited CIH-induced ER stress in the liver, as evidenced by decreased expression of BiP, unfolded protein response transducers, and ER proapoptotic proteins.
**Cai XH, et al. [70]**	OSA (CIH)	Animal model study (case-control)	Rat	qPCR, Western blotting	BiP, ATF4, ATF6, XBP-1, CHOP	Apoptosis was increased and phosphorylation of PERK and IRE1 was upregulated in CIH groups. Sal prevented activation of CHOP throughout hypoxia/reoxygenation exposure.
**Perrini S, et al. [71]**	OSA (CIH)	Case-control study	Human	RT-PCR	ATF4, CHOP, ERO-1	Adipose tissue mRNA levels of ER stress markers (ATF4, CHOP, ERO-1) were decreased only in the therapeutic CPAP group compared with non-OSA and subtherapeutic CPAP groups.

ER, endoplasmic reticulum; AR, allergic rhinitis; HDM, house dust mite; p-IRE, phosphorylation of inositol-requiring enzyme; GRP, glucose-regulated protein; BiP, immunoglobulin heavy chain-binding protein; ERp, endoplasmic reticulum protein; ATF, activating transcription factor; CHOP, C/EBP-homologous protein; CRSwNP, chronic rhinosinusitis with nasal polyp; p-eIFα, phospho-eukaryotic initiation factor-α; SEB, *S. aureus* enterotoxin B; ROS, reactive oxygen species; EP, eosinophilic polyp; NEP, non-eosinophilic polyp; p47^phox^, neutrophil cytosol factor 1 (NCF1); NP, nasal polyp; IT, inferior turbinate of the nose; PNEC, primary nasal epithelial cells; RT-PCR, reverse transcription-polymerase chain reaction; XBP1, X-box-binding protein 1; TGF, tumor growth factor; EMT, epithelial-mesenchymal transition; α-SMA, alpha-smooth muscle actin; 4-PBA, 4-phenylbutylic acid; PP2, Src and RIP2 kinase inhibitor; OSA, obstructive sleep apnea; CIH, chronic intermittent hypoxia; PERK, endoplasmic reticulum kinase; Ad, adiponectin; LVF, left ventricular function; TUNEL, terminal deoxynucleotidyl transferase dUTP nick-end labeling; Bax, BCL2-associated X; PAG, DL-propargylglycine; HIT, high intensity training; HIF-1α, hypoxia inducible factor-1α; TUDCA, tauroursodeoxycholic acid; ELISA, enzyme-linked immunosorbent assay; JNK, c-Jun N-terminal kinase; qPCR, quantitative polymerase chain reaction; Sal, salubrinal; ERO-1, endoplasmic reticulum oxidoreductin-1; CPAP, continuous positive airway pressure.
